# Psychometric Properties of the Malay-Language Quality of Life Enjoyment and Satisfaction Scale: A Confirmatory Study on Malaysian Children

**DOI:** 10.3390/ijerph18020622

**Published:** 2021-01-13

**Authors:** Mohamad Fadil Ibrahim, Garry Kuan, Hairul Anuar Hashim, Nurul Azuar Hamzah, Yee Cheng Kueh

**Affiliations:** 1 Exercise and Sports Science, School of Health Sciences, Universiti Sains Malaysia, Kubang Kerian 16150, Kelantan, Malaysia; fadzil@ipgmksm.edu.my (M.F.I.); hairulkb@usm.my (H.A.H.); nazuar@usm.my (N.A.H.); 2 Institut Pendidikan Guru Malaysia, Kampus Sultan Miza, Besut 22200, Terengganu, Malaysia; 3 Department of Life Sciences, Brunel University, London UB8 3PH, UK; 4 Biostatistics and Research Methodology Unit, School of Medical Sciences, Universiti Sains Malaysia, Kubang Kerian 16150, Kelantan, Malaysia; 5 Psychiatry Department, School of Medical Science, Universiti Sains Malaysia, Kubang Kerian 16150, Kelantan, Malaysia

**Keywords:** confirmatory factor analysis, physical health, feelings, homework, leisure time

## Abstract

The objective of this study was to validate the translated Malay version of the Quality of Life Enjoyment and Satisfaction Questionnaire (Q-LES-Q-M) scale among Malaysian primary school children using the confirmatory factor analysis (CFA). The Q-LES-Q-M measures the level of enjoyment and satisfaction experienced with relation to physical health, feelings, homework, and leisure. The participants were 607 Malay students, 240 (39.5%) boys and 367 (60.5%) girls, with an age range from 10 to 11 years old. The original version of the Q-LES-Q was translated into the Malay language by forward to backward translation procedures with consideration for the local culture and suitable vocabulary for primary school students. The participants then completed the Q-LES-Q-M. CFA was performed using Mplus 8 software. Using CFA, the initial model did not result in a good data fit. Further analysis of the CFA suggested some changes to the model to improve the fit indices. Model modification included the deletion of three problematic items and co-varying some error items. This resulted in improved fit indices and 40 items remained in the final model. The final model showed good reliability based on two indicators of composite reliability (CR) and Cronbach’s alpha (CA). The factors with their CR and CA were physical activity (CR = 0.857, CA = 0.854), feelings (CR = 0.808, CA = 0.813), homework (CR = 0.837, CA = 0.837) and leisure (CR = 0.742, CA = 0.737). The final measurement consists of 40 items. The retained items were deemed suitable for Malay primary school children. The revised Q-LES-Q-M with 40 items is suitable for measuring the levels of children’s involvement in determining the enjoyment and satisfaction of learning and physical activity.

## 1. Introduction

Students with balanced physical, emotional, spiritual, intellectual, and social needs have been shown to have a higher level of self-discipline [1,2]. These balanced components of the self can be achieved via participation in games and physical activity [3]. Interactions during play allow children to develop social skills with respect to sharing and teamwork, as well as the pleasure of learning and exploring through play activities, which will stimulate their creativity [4,5]. In addition, play makes children learn to solve cognitive, motor, and social problems [6] that will develop their social and emotional competence [7]. It also allows students to learn to solve a variety of problems (cognitive, motor, and social) that contribute to their intellectual development [4,8]. Regular participation in physical activity can reduce the risk of depression [9] and can prevent the spread of diseases such as hypertension, obesity, cardiovascular disease, and diabetes [10,11].

The Quality of Life and Satisfaction Questionnaire (Q-LES-Q) is an integrated model that assesses behavioral changes related to quality of life (QOL) based on a combination of various psychological theories [12,13]. The entire original Q-LES-Q questionnaire consisted of eight constructs consisting of physical health, feelings, work, household duties, school/homework, leisure time activities, social relations, and general activities. Researchers have identified four relevant constructs from the eight constructs found in the Q-LES-Q, namely physical health, feelings, homework, and leisure. The Q-LES-Q can identify the extent to which pleasure and satisfaction, as assessed through specific observations about physical health, feelings, homework, and leisure time, will affect changes in student behavior, especially regarding QOL [12]. The Q-LES-Q has been widely used in testing various behaviors both emotionally as well as health and physical aspects that transcend various ages [12,14].

Research related to enjoyment and student satisfaction is seen to be lacking in the literature, especially in Malaysia. Considerations were made through the selection of the four main domains of the Q-LES-Q that relate explicitly to student enjoyment and satisfaction, enabling researchers to identify QOL and relationships with students’ physical activities that are lacking in Malaysian society. Therefore, there is a need for researchers to have a validated scale for measuring enjoyment and satisfaction among children in doing physical activity in Malaysia. The translations of the Q-LES-Q from English to Malay fill the gap and can serve as a useful tool to identify the level of enjoyment and satisfaction of primary school students, but also assist the function of the Malay language itself as the lingua franca [15], especially in Southeast Asia, in which further research is needed. To this end, researchers are aware of the need to identify and find more appropriate forms of measurement to assess QOL and physical activity. The Q-LES-Q is seen to assess different dimensions, such as integrated physical health, feelings, exercise, and leisure activities, which are found in the questionnaire [14]. The Q-LES-Q was developed to identify and evaluate various important aspects of a child and adolescent’s life experience, and it has been tested for validity and reliability [12,14].

The Q-LES-Q is a measure of how sensitive participants are to the enjoyment and satisfaction of different daily activities, especially with regards to children’s involvement in physical activities that will affect their quality of life [16]. Therefore, this study is important given the fact that the Q-LES-Q was seldom used for the younger population. Providing a valid instrument measuring these important constructs among children is timely and important. Thus, the purpose of this study is to translate the Q-LES-Q into the Malay language to be used among the Malaysian population and, in turn, to examine the reliability and validity of the translated Malay version of the Q-LES-Q (Q-LES-Q-M) among the primary school students.

## 2. Materials and Methods 

### 2.1. Study Design, Recruitment, and Sampling

The research employed a cross-sectional study design. The data were collected between May 2019 through October 2019, among ten primary schools, in the district of Besut, Terengganu, Malaysia. 

### 2.2. Participants

A total of 623 Standard 4 and 5 students were randomly chosen from 10 participating schools using a computer-generated random number. The inclusion criteria included participants who were children aged between 10 and 12 years old and were able to read and complete the Malay version questionnaire. From the total 623 questionnaires returned, 16 questionnaires were incomplete and thus were discarded from the data analysis. Therefore, the response rate based on completed questionnaires was 97%. Based on Hair et al. [17], the sample size for confirmatory factor analysis (CFA) requirements may exceed 500. In the present study, the Q-LES-Q-M consists of four subscales, so the sample size of 607 was sufficiently large for the present confirmatory study to use CFA. 

### 2.3. Measures

#### 2.3.1. Demographic Form 

Some questions relating to the background of physical activity and leisure are addressed in the form of demographics. In addition, questions assessing the participants’ backgrounds including age, gender, and ethnicity, and participants’ involvement in physical and leisure activities within the previous week were also included. 

#### 2.3.2. Quality of Life, Enjoyment and Satisfaction Questionnaire

The original Q-LES-Q questionnaire was developed by Endicott et al. [12]. This questionnaire is designed to determine the level of enjoyment and satisfaction experienced by outpatients in various areas of daily functioning. The full version of the Q-LES-Q was selected rather than the short version of the PQ-LES-Q because we aimed to assess students’ enjoyment and satisfaction based on multiple domains rather than a single domain or general assessment. The selected domains from the Q-LES-Q questionnaire for the present study included physical health (13 items), feelings (14 items), homework (10 items), and leisure time (6 items) with a total of 43 items. Preliminary evidence of the reliability and validity of the pediatric sample on the PQ-LES-Q suggests that this questionnaire was found suitable to be used in children and adolescents as a QOL measurement [13]. The questionnaire was measured on a 5 point Likert scale consisting of: (1) not at all, (2) rare, (3) sometimes, (4) frequent, and (5) all the time. The respondents were asked to respond to the items based on their priorities. The Q-LES-Q shows good reliability based on a validation study conducted by Rossi et al. in a study conducted in Italy in five Italian academic centers (Pisa, Florence, Torino, L’Aquila, Siena) on outpatients who met DSM-IV criteria for anxiety disorder. Internal consistency based on Cronbach’s alpha shows a range of between 0.88 to 0.96, and the reliability of tests based on intra-class correlations ranged from 0.80 to 0.97 [18]. This indicated that the Italian version of the Q-LES-Q for adults was reliable and stable across the time. The Q-LES-Q was translated into the Malay language and validated in the present study. 

### 2.4. Questionnaire Translation 

In the present study, four domains (i.e., work, household duties, social relations, general activities) from Q-LES-Q were excluded due to the content of the items not being suitable for the study population. The remaining four domains of the Q-LES-Q (with 43 items) were then translated into the Malay language using the standard forward and backward translation procedure recommended by Brislin [19]. Two content experts checked the English translation into the Malay language, ensuring the translation made by the researchers retained the original meanings and was suitable for children. Next, a local bilingual Malay lecturer back-translated the Malay version to English. Then, three lecturers and two teachers with experience in sports psychology, sports science and physical education reviewed the English translation from Malay and the Malay translation of English and compared each item to the matching original English version. All five panels had over 10 years of experience in their areas of expertise and were competent bilingual speakers of Malay and English. They also evaluated whether the content was consistent with Malaysian culture, suitable for Malaysian children aged above 10 years old and ensured that it did not deviate from the original meaning and purpose of the survey. This is a common approach used by researchers when translating a questionnaire from a different language into the Malay language [20,21,22]. A pilot test of the final version the Q-LES-Q-M was conducted among 30 primary school students in Besut, Terengganu. They were asked to answer the questionnaire. The results of the pilot test showed that the Cronbach alpha for the translated Q-LES-Q-M showed high values (physical health 0.81, mood 0.72, homework 0.74 and leisure time 0.64) and was suitable to be applied to the target group. The Q-LES-Q-M is available from the authors on request. 

### 2.5. Procedures

The research was performed in compliance with the Declaration of Helsinki and was approved by the Universiti Sains Malaysia Human Research Ethics Committee (USM/JEPeM/19090518). Approval was also obtained from the Ministry of Education of Malaysia, Terengganu State Education Department, and the principals of the respective schools. All participants were given consent and assent forms for their parents to read, comprehend and sign to allow their children to participate in this study. In addition, the research information sheet was also provided. The students were told that their participation was voluntary and that they could withdraw from the study at any time without any loss of the benefits to which they were entitled. Questions relating to personal identifiers were not included in the questionnaire, which is commonly applied in other studies involving questionnaires [21,23]. The questionnaire was administered to participants during school hours. Before the questionnaire was distributed, the questionnaire was clearly explained to them. The participants completed the questionnaire via Google Forms in the school computer lab with the aid of teachers. They took an average of 20 min to complete the questionnaire. Once they finished, the students were given a certificate as a token of appreciation for participating in the study. 

### 2.6. Data Analysis

Descriptive statistics and internal consistency based on the Cronbach alpha value were conducted using SPSS 25.0. CFA was tested using Mplus 8. The assumption of multivariate normality was checked using Mplus and was not met (Mardia multivariate skewness and kurtosis test with *p*-values less than 0.05). Therefore, the MLR estimator was then used in the subsequent CFA analyses.

The initial hypothesized measurement model consists of 4 latent variables (i.e., physical health, feelings, homework, and leisure time) and 43 observed variables (items in the Q-LES-Q-M). Factor loadings of 0.40 and above, with significant *p*-values and modification indices, were used as a guide to retain or remove items from the measurement model [24]. The evaluation of model fit was carried out each time the model was re-specified or a problematic item was removed. Multiple fit indices were used to decide whether to reject or retain a model [17,25]. The fit indices that were used and the recommended fit values were: the comparative fit index (CFI) and Tucker and Lewis index (TLI) with the desired value of more than 0.92, the root mean square error of approximation (RMSEA) with the desired value of less than 0.07 and Close fit p-value greater than 0.05, and the standardized root mean square (SRMR) with the desired value of less than 0.08 [17,25]. After the best-fit measurement model was established, the four factors were assessed for composite reliability (CR) and discriminant validity. The CR was calculated based on Raykov’s method to measure the reliability of latent variables in the CFA measurement model [26]. The minimum acceptable value of CR is 0.60 and above [17,27]. Discriminant validity was used to analyze the degree to which one factor is distinct from the other factors [25]. If the correlation coefficient between factors is not too high ≤0.85, then the validity of discrimination can be identified [25].

## 3. Results

The total number of respondents was 607 participants: 240 (39.5%) were male, and 367 (60.5%) were female. When considering respondents by age categories, a total of 186 (30.6%) respondents were aged 10 years and 421 (69.4%) respondents were aged 11 years. All participants were Malay. 

### 3.1. Measurement Model for the Q-LES-Q-M

The hypothesized Q-LES-Q-M measurement model consists of 4 factors with 43 items, namely physical health (13 questions), feelings (14 questions), homework (10 questions), and leisure time (6 questions). Three items were found to be problematic as they resulted in low factor loading or caused a reduction in the goodness-of-fit. The results of all models tested are shown in Table 1 and Table 2. The initial model (Model 1) did not fit the data well based on several fit indices (see Model 1, Table 1). There were three items (P2, P8, and L10) with a factor loading of less than 0.40. The contents of the items were analyzed, and we found that removing the items from the construct would not have an affect the theoretical concept of the Q-LES-Q-M. After removing the problematic items, the fit indices of the measurement model have been improved (see Model 2, Table 1). 

Again, the improvement in fit indices was not considered to be sufficient. The modification index suggested that adding some covariance between the items’ error terms would improve the model’s fitness. Adding the covariance between these items’ errors seems rational, as both items were within their own factor and had similar content. After incorporating the covariance between the error terms for items F7 (felt you were getting enough sleep) and F2 (felt rested), F5 (felt very good in physical health) and F4 (felt in excellent physical health), the model resulted in a good fit to the data (see Table 1, Model 3). The values of fit indices and the factor loadings of the final model were presented in Table 1 and Table 2.

### 3.2. Reliability and Discriminant Validity

Based on the final model, the CR was computed, and values ranged from 0.74 to 0.84, suggesting moderate to good construct reliability. The Cronbach alpha of each factor ranged from 0.74 to 0.85. The Cronbach alpha and CR values for all constructs indicate satisfactory reliability of the construct. The correlation values between the four-factor ranged from 0.21 to 0.28. All correlations were below the recommended value of 0.85, which suggests the good validity of discrimination. The values of the CR, Cronbach alpha, and correlation coefficients are shown in Table 3. 

**Figure 1:**
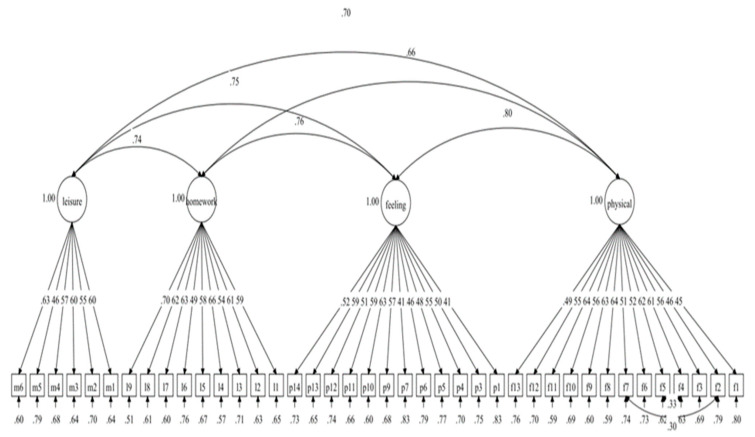
The confirmatory factor analysis (CFA) diagram for the final measurement model of the Q-LES-Q-M.

The final model (Model 3) was established by deleting three items (P2, P8, and L10) and adding a covariance between the items’ errors for items (F7, F2) and (F5, F4) (see Figure 1). Figure 1 illustrates the final measurement model of the Q-LES-Q-M. 

## 4. Discussion

The Q-LES-Q has been widely translated and used to measure how behavior in determining enjoyment and satisfaction in four key areas, namely physical health, mood, homework, and leisure time related to physical activity in different study populations. For validation studies, the study aimed to verify whether the four-factor item measurement model consisting of 43 items could be well adapted. The CFA analysis results confirm the validity and reliability of the revised Q-LES-Q-M following the CFA analysis being changed to 40 items. Based on this validity and reliability study, the revised version of the Q-LES-Q-M with 40 items is considered suitable to be used in primary school students aged 10 and 11 years old.

Based on empirical studies related to enjoyment and satisfaction in assessing students’ QOL [13,28,29] as well as involvement in physical activity, the Q-LES-Q is very suitable to be used as a measure of quality of life enjoyment and satisfaction, which is a wider scope than other questionnaires because the Q-LES-Q questionnaire has been widely tested worldwide [14,18,30,31]. Thus, the researchers translated part of the Q-LES-Q for the needs of local students in Malaysia to see the relationship between enjoyment and satisfaction with the physical activity and QOL. The questionnaire was translated into Malay because Malay is the national language.

The confirmation of the Q-LES-Q is important in determining the level of enjoyment and satisfaction of individuals, particularly among the Malaysian community. In this study, the researchers conducted a factor validation of the Q-LES-Q-M structure. The full version of the Q-LES-Q has been shown to be reliable, valid, and stable over time, based on previous studies [14,18]. Researchers translating part of the Q-LES-Q to the needs of local students allowed this study to identify the validity and reliability models needed to produce consistent questionnaires, showing that good value requires greater modeling by discarding items with low factor loading [16]. Most of the previous studies on the Q-LES-Q have shown good model performance for students at higher education institutions, but not in primary school students [13,14,29,31]. In this study, the items were tailored to the needs of the students in primary school. 

The first level of items with low loading factor value (items P2, P8, and L10) was excluded. After some investigation, we found that these low loadings may be due to the lack of relevance of the questions to study participants who were younger in age. For example, item P2 is about the satisfaction of life. This question is more appropriate for older participants, who have more experience and a better understanding of self-satisfaction regarding their own life. Therefore, the item was appropriate to be omitted from the measurement model. 

This current study retained 40 items in the final measurement model, after removing items with low loading factor values. The CFA analysis of the studies on the Q-LES-Q-M supports the four-factor structure proposed. Moreover, it shows a high internal validity of the sample being studied. The Cronbach alpha obtained from the present study ranged from 0.74 to 0.85, which was quite similar to other Q-LES-Q validation studies [14,18]. A parsimonious subset of items from the Q-LES-Q that can accurately predict the mean score of the Q-LES-Q domain were accurately assessed in 339 patients who meet the DSM-IV criteria for schizophrenia, schizoaffective disorder, and mood disorders, showing that the questionnaire has good reliability and validity when used to measure patient quality of life in overcoming anxiety [29]. The Chinese version of the Q-LES-Q-SF study by Lee et al. also showed a high-reliability value of 0.911. Besides, this study also reports that the value of reliability based on the CR and the CR value is ≥0.7, which indicates a satisfactory reliability [30]. Ritsner et al. reported that the Cronbach’s alpha value for the Q-LES-Q-18 short version was high based on a patient sample (0.82–0.94) and healthy subjects (0.74–0.88) [29]. These high Cronbach’s coefficient values were consistent with the present study.

The strength of this study included a large sample size (607 respondents). Assessing a child’s QOL and relationships with physical activity is an important indicator of well-being that is valuable for assessing student self-satisfaction. The researchers’ goal is to help improve the QOL and good personal health through this construct. Recent results provide further evidence of the reliability and validity of the Q-LES-Q-M for use in children and adolescents but warn other researchers not to use the Q-LES-Q-M as a self-report measure for children under 10 years. Future studies should examine the Q-LES-Q-M’s reliability in a more diverse sample, including a wider student population. Further studies are also expected to build a better quality of life questionnaire by considering the child’s developmental stage. However, there are some limitations to this study that researchers should acknowledge. First, this study was conducted only in one district and randomly selected schools may not reflect the entire population of Malaysia, as well as different ethnic and socio-cultural backgrounds. The second limitation is that the administration of the questionnaire via Google Forms increases the respondents’ carelessness and feedback in choosing the right answer even though the focus-related problem was addressed. In this context, the researchers emphasized the importance of honest feedback to the subjects prior to data collection. As the questionnaire was answered online, the opportunity for respondents to respond in a socially acceptable manner was reduced. The third limitation is that other validity tests, such as concurrent validity test, were not included in this study. In future, a similar, established questionnaire should be used to correlate its results with the translated version of the Q-LES-Q. If the correlation is satisfactory, this will further support the validity of the questionnaire. 

## 5. Conclusions

This study showed that the original English version of the Q-LES-Q could be successfully adapted to Malay with satisfactory psychometric properties of validity and reliability among primary students in Malaysia. Previously the only available questionnaires Malay were limited and unreliable for measuring the level of enjoyment and satisfaction experienced by students in Malaysia. Therefore, it is very important to have a version of the Q-LES-Q in the Malay language, and its accurate translation and reliability is important. The experience of enjoyment and satisfaction is an important aspect of life. In this study, the final measurement model for the Q-LES-Q-M questionnaire consisted of 40 items. Most of the items were retained, and items were considered appropriate for the sample in this study. This instrument is considered valid because the questions are suitable for testing students in relation to QOL, to the extent to which a person experiences enjoyment and satisfaction. Researchers, sports psychologists, and Physical Education teachers who might use the Q-LES-Q-M would find this questionnaire to be a useful and simple tool that can be applied routinely in measuring and monitoring the psychological impact on QOL-related levels of pleasure and satisfaction among primary school students in Malaysia. Therefore, improvements can be made through strategies to increase enjoyment and satisfaction, and thus to increase the physical activity of the Malaysian population and reduce the increase in various diseases in the Malaysian population. If it can be well integrated, it may be a useful adjunct in the daily practice in QOL awareness of this important aspect of life.

## Figures and Tables

**Table 1:** A summary of the models’ fit indices.

Path Models	RMSEA (90% CI)	RMSEA *p*-Value	CFI	TLI	SRMR
Model 1	0.031(0.031, 0.037)	0.637	0.896	0.890	0.046
Model 2 ^a^	0.032 (0.029, 0.035)	0.972	0.914	0.908	0.044
Model 3 ^b^	0.029 (0.025, 0.032)	1.000	0.930	0.926	0.042

^a^ The measurement model with P2, P8, and L10 deleted, ^b^ The measurement model with P2, P8, and L10 deleted and covariance between the item errors of (F7 with F2) and (F5 with F4); RMSEA = root mean square error of approximation; CI = confidence interval; CFI = comparative fit indices; TLI = Tucker and Lewis index; SRMR = standardized root mean square.

**Table 2:** The standardized factor loadings for model 1, model 2, and model 3.

Factors and Items	Factor Loadings		
	Model 1	Model 2	Model 3
Physical			
F1	0.445	0.445	0.448
F2	0.484	0.484	0.461
F3	0.563	0.563	0.561
F4	0.639	0.639	0.607
F5	0.647	0.647	0.617
F6	0.520	0.520	0.519
F7	0.520	0.520	0.506
F8	0.636	0.636	0.640
F9	0.626	0.626	0.629
F10	0.553	0.533	0.558
F11	0.636	0.635	0.641
F12	0.543	0.542	0.550
F13	0.491	0.490	0.492
Feelings			
P1	0.411	0.413	0.412
P2	0.267	-	-
P3	0.511	0.502	0.502
P4	0.544	0.544	0.545
P5	0.481	0.484	0.483
P6	0.467	0.462	0.461
P7	0.422	0.412	0.412
P8	0.377	-	-
P9	0.568	0.569	0.569
P10	0.626	0. 630	0.530
P11	0.586	0.586	0.586
P12	0.510	0.507	0.508
P13	0.584	0.591	0.591
P14	0.516	0.517	0.517
Homework			
L1	0.587	0.595	0.595
L2	0.600	0.608	0.608
L3	0.534	0.537	0.537
L4	0.622	0.659	0.659
L5	0.580	0.578	0.578
L6	0.494	0.493	0.492
L7	0.632	0.633	0.633
L8	0.624	0.625	0.625
L9	0.697	0.700	0.700
L10	0.327	-	-
Leisure time			
M1	0.598	0.597	0.597
M2	0.551	0.552	0.551
M3	0.597	0.599	0.599
M4	0.571	0.570	0.570
M5	0.463	0.462	0.462
M6	0.633	0.634	0.634

**Table 3:** Composite reliability (CR), Cronbach’s alpha (CA) and the factor correlation of the final model for the Q-LES-Q-M.

Variable	CR	Cronbach Alpha	1	2	3	4
1. Physical	0.84	0.85	1	0.22	0.25	0.24
2. Feelings	0.81	0.81		1	0.23	0.21
3. Homework	0.84	0.84			1	0.28
4. Leisure time	0.74	0.74				1

Correlation is significant at the 0.05 level (two-tailed); CR = composite reliability.

## Data Availability

The data is available upon request from the authors.
